# CD200 is a potent new growth factor in Multiple Myeloma dependent on IGF1 receptor signaling

**DOI:** 10.1016/j.tranon.2026.103000

**Published:** 2026-08-28

**Authors:** Angélique Bruyer, Nicolas Robert, Jules Varbedian, Elvira Garcia De Paco, Lauryn Jaffory, Stéphanie Boireau, Christophe Hirtz, Jérôme Vialaret, Pierre Martineau, Bruno Robert, Guilhem Requirand, Guillaume Cartron, Laure Vincent, Charles Herbaux, Jérôme Moreaux, Caroline Bret

**Affiliations:** a Institut de Génétique Humaine, Univ Montpellier, CNRS, Montpellier, France; b Department of Biological Hematology, St Eloi Hospital, Montpellier University Hospital, Montpellier, France; c IRMB-PPC, INM, Univ Montpellier, CHU Montpellier, INSERM CNRS, Montpellier, France; d IRCM, University of Montpellier, ICM, INSERM, Montpellier, France; e Department of Hematology and Clinical Oncology, St Eloi Hospital, Montpellier University Hospital, Montpellier, France; f University of Montpellier, Faculty of Medicine, 34090 Montpellier, France; g Institut de Génétique Moléculaire de Montpellier, University of Montpellier, CNRS-UMR 5535, Montpellier, France; h Institut Universitaire de France, Paris, France

**Keywords:** Multiple myeloma, CD200, IGF1R, Growth factor, AKT

## Abstract

CD200 is an immune checkpoint aberrantly expressed in 70% of newly diagnosed patients with multiple myeloma (MM) and associated with a poor outcome. CD200 expression by tumor cells inhibits immune response through interaction with CD200R and participates in resistance to immune based therapies. However, its role in tumorigenesis remains poorly understood. In this study, we show that MM cells overexpressing CD200 present a significant proliferative advantage in serum free culture medium and clonogenic assays. The cell growth advantage mediated by CD200 in MM cells is dependent on the presence of IGF1R but independent on the presence of CD200R. Of interest, we reported that high expression of *CD200* and *IGF-1R* is associated with a poor outcome in two independent cohorts of MM patients. Moreover, immuno-precipitation and mass spectrometry experiment identifies CD200 in the IGF1R-associated protein complex in association with AKT phosphorylation at Threonine 308 and activation. In conclusion, we demonstrate, for the first time, that CD200 supports myeloma cell growth through interaction with IGF-1R. Furthermore, combined approaches targeting CD200 and IGF-1R may provide new therapeutic avenues to target CD200 mediated cell growth function and its immunosuppressive functions associated with resistance to immune based therapies in MM.

## Highlights

• CD200 acts as a myeloma growth factor in Multiple Myeloma (MM) cells.
• The cell growth advantage mediated by CD200 in MM cells is dependent on the presence of IGF1R but independent on the presence of CD200R.
• CD200 is a component of the IGF1R-associated protein complex and is linked to AKT phosphorylation and activation.

## Introduction

CD200 is a highly conserved transmembrane glycoprotein acting as an immune checkpoint molecule. Belonging to the immunoglobulin superfamily, it is widely expressed by various normal cell types, including thymocytes, B cells, activated T cells, resting dendritic cells, endothelial cells or neurons [1,2]. CD200 is also expressed by cancer cells, during hematological malignancies [3], malignant melanoma [4] or neuroendocrine cancers [5].

CD200 interacts with its inhibitory receptor CD200R, to exert its canonical action leading to immunosuppressive effects. Several isoforms of this receptor have been reported [6], the most known being CD200R1, which is expressed on cells of the monocyte/myeloid lineage and by some T cell subsets [[7], [8], [9]]. CD200R is also highly expressed in the tumor microenvironment, at the surface of tumor-associated macrophages, myeloid-derived suppressor cells and tumor-associated dendritic cells [10].

Following the CD200/CD200R interaction, intracellular adaptor proteins are recruited, as well as other partners as SHIP and Ras GAP, to finally inhibit Ras/MAPK signaling pathway. Non-canonical effects of CD200 were also described, promoting a pro-tumor phenotype, independently of signaling through the receptor CD200R [2].

We previously identified CD200 as a prognostic factor in multiple myeloma (MM), a hematological malignancy characterized by the accumulation of malignant plasma cells (MMCs) within the bone marrow environment. *CD200* gene expression was identified in MMCs of 78% patients as initially reported by our group and then confirmed by other studies [[11], [12], [13]]. CD200 expression is associated with a shorter progression-free survival in MM patients treated by high dose melphalan and autologous stem cell transplantation [11] or anti-CD38 monoclonal antibody (MoAb) [14].

CD200^+^ MMCs displayed a reduced immunogenicity *in vitro* [15]. In addition, CD200 expression level at the surface of MMCs was correlated to the expansion of regulatory T cells and to the level of serum cytokines including interleukin 6, interleukin 10 and Transforming Growth Factor beta [16]. However, with the exception of the immunosuppressing effects of the CD200/CD200R axis, little is currently known about biological functions of CD200 in MM. In a murine model characterized by the absence of CD200R, tumor-associated macrophages will present deregulation of several chemokines including upregulation of CCL24 and CCL8 associated with increased infiltration of neutrophils together with higher anti-tumor activity [17]. Shedding of CD200 by MMP3 and MMP11 metaloproteinase is associated with release of active soluble CD200 [18]. In mouse xenograft, soluble CD200 could suppress MAPK pathway signaling in NK cells, through CD200R, leading to NK cell dysfunction and apoptosis [18]. More recently, CD200 expression was shown to suppress anti-BCMA CAR-T cell cytotoxicity in MM models [19].

Non canonical mechanism related to CD200 biological functions and unrelated to CD200R engagement was reported [20]. The 19-aa cytoplasmic tail of CD200 is a target for cleavage by γ-secretase, releasing a tail fragment capable of nuclear translocation and DNA binding. The nuclear fragment binds DNA and increases expression of transcription factors associated with cell growth in B cell lines and fresh patient CLL cells [20]. This study suggested a mechanism related to a biological effect of the cytoplasmic domain of CD200 that support activation of transcription factors and cellular growth in leukemic cells [20].

Several autocrine or paracrine growth factors promote MMCs survival, proliferation and homing, including interleukin-6 [21], insulin-like growth factor 1 [22], insulin [23], epidermal growth factor family members [24], B cell activating factor (BAFF), a proliferation inducing ligand (APRIL) [25], hepatocyte growth factor or stromal cell-derived factor 1 [26]. In this study, we demonstrate for the first time that CD200 induced myeloma cell growth *via* an association with the receptor of insulin-like growth factor 1 (IGF1R) and AKT phosphorylation at Threonine 308.

## Materials and methods

The ethic board of the University Hospital of Montpellier approved the study.

### Human myeloma cell lines (HMCLs)

XGs cell lines were obtained as previously described [[27], [28], [29]]. AMO-1, LP1, L363, SKMM2, KMS27 MOLP8 and OPM2 were purchased from DSMZ (Braunsweig, Germany) and RPMI8226 from ATCC (Rockville, MD, USA). JJN3 was kindly provided by Dr. Van Riet (Bruxelles, Belgium). These cell lines were maintained in RPMI1640 GlutaMAX (61,870,044, Gibco) supplemented with 10% fetal bovine serum (CVFSVF0001, Eurobio), and with IL-6 (2ng/ml) for the XGs. HMCLs were authenticated according to their short tandem repeat profiling and their gene expression profiling using Affymetrix U133 plus 2.0 microarrays deposited in the ArrayExpress public database under accession numbers E-TABM-937 and E-TABM-1088.

### Plasmids, lentivirus and transduced cell lines

VSV-G lentivirus were produced using Invitrogen methodology with the human HEK 293T cell line, Dulbecco’s modified Eagle’s medium supplemented, Vira-Power-packaging mix and plasmid of interest (pLenti6.3_CD200, pLenti6.3_GFP or pLenti4_shCD200R (Invitrogen, Carlsbad, USA) as previously described [30,31]. Overexpression of CD200, GFP or depletion of CD200R in HMCLs were validated by flow cytometry. Transduced cell lines were used for assays if the viability was superior to 90%.

For studies involving shCD200R, HMCLs were first transduced with a lentiviral vector containing the tretracycline repressor (TR) before transduction with a lentivirus containing a shCD200R under the control of TetO (inductible expression by doxycycline). LP1_TR_shCD200R was also co-transduced by a lentiviral vector containing CD200 gene with a constitutive expression. The shCD200R were provided by Thermo Fisher Scientific “CD200R1 BLOCK-IT miR Hmi455684” with sequence 5′TGCTGAACAGTAGCCCTAGGTTAGCAGTTT3’ and 5′CCTGAACAGTAGCCCGGTTAGCAGTCAGTC3’.

### Assay and reagents for myeloma cell line growth

All HMCLs were serum and IL-6- starved for 2 h with SYNH medium as previously described [22] and cultured for 4 days in 96-well flat-bottom microtiter plates in Syn-H serum-free culture medium with or without cytokine or monoclonal antibody. HMCL cell growth was evaluated by quantifying intracellular ATP amount with a Cell Titer Glo Luminescent Assay (Promega, Madison, WI, USA) with a Centro LB 960 luminometer (Berthold Technologies, Bad Wildbad, Germany).

The survival and proliferative advantage of CD200 positive cells, was also tested using a co-culture mix assay. On day 0, 50% of GFP-positive cells and 50% of CD200-positive cells were mixed in the same well. On day 0, 7 and 10, the percentage of GFP in the culture cell was determined by flow cytometry.

Control IgG1, CD200 and IGF-1R MoAbs (clone 11,711, clone 325,531 and clone 33,255 respectively, R&D, Minneapolis, MN, USA) have been used. In cell growth experiments, recombinant IGF-1 (R&D, Minneapolis, MN USA), recombinant insulin (Roche Diagnostics, Basel, Switzerland) or fetal calf serum (FCS, PAA laboratory GmbH, Linz, Austria) were added on day 0. In shCD200R experiments, doxycycline (1 mg/ml, Sigma, St Louis, MO, USA) was added on day 0 and day 4.

### Clonogenic assay

Ten thousand cells were plated into a 35 mm tissue culture plate for layer agar cultures. Cells were re-suspended in 33% collagen solution and in Stem Alpha III medium according to the manufacturer’s instructions (Stem alpha III, Saint Genis L’argentière, France). Cells were fed twice a week by placing two drops of medium on the layer with or without monoclonal antibody or IL-6 for IL-6-dependent cell lines during 3 weeks. Colony number was enumerated.

### RNA-sequencing

Bone marrow of patients presenting with previously untreated MM at the university hospital of Montpellier was obtained after patients' written informed consent in accordance with the Declaration of Helsinki and agreement of IRB and the Montpellier University Hospital Centre for Biological Resources (DC-2024–6611). Patients’ MM cells (MMCs) were purified using anti-CD138 MACS microbeads (Miltenyi Biotec, Bergisch Gladbach, Germany) and their gene expression profile (GEP) obtained using RNA-sequencing. The RNA sequencing (RNA-seq) library preparation was done with 150 ng of input RNA using the Illumina TrueSeq Stranded mRNA Library Prep Kit. Paired-end RNA-seq was performed with an Illumina NextSeq sequencing instrument (Helixio, Clermont-Ferrand, France). RNA-seq read pairs were mapped to the reference human GRCh37 genome using the STAR aligner. The RNA sequencing (RNA-seq) library preparation was done with 300 ng of input RNA using the Illumina TruSeq Stranded mRNA Library Prep Kit. Paired-end RNA-seq were performed with Illumina NEBNext Ultra II Directional RNA library Prep kit (Integragen, Evry, France). RNA-seq read pairs were mapped to the reference human GRCh37 genome using STAR (Gentleman RC et al., Genome Biol., 2004). All statistical analyses were performed with the statistics software R (available from https://www.r-project.org) and R packages developed by BioConductor project (available from https://www.bioco nductor.org/) (Gentleman RC et al., Genome Biol., 2004). The expression level of each gene was summarized and normalized using DESeq2 R/Bioconductor package. Differential expression analysis was performed using DESeq2 pipeline. p values were adjusted to control the global FDR across all comparisons with the default option of the DESeq2 package. Pathway enrichment analyses were performed using online curated gene set collection on the Gene Set Enrichment Analysis software (https://www.gsea-msigdb.org/gsea/index.jsp). RNA sequencing data of normal memory B cells, normal preplasmablasts, normal plasmablasts and normal plasma cells generated as described [32]. Data are available in Gene Expression Omnibus under the accession number GSE148924. We used the Maxstat R function for unbiased dichotomization and determine the optimal cutpoint for continuous variables [33] as previously reported [34,35].

Supplementary information is included in Supplemental Methods.

## Results

### CD200 and CD200R expression in multiple myeloma cells

**Fig. 1:**
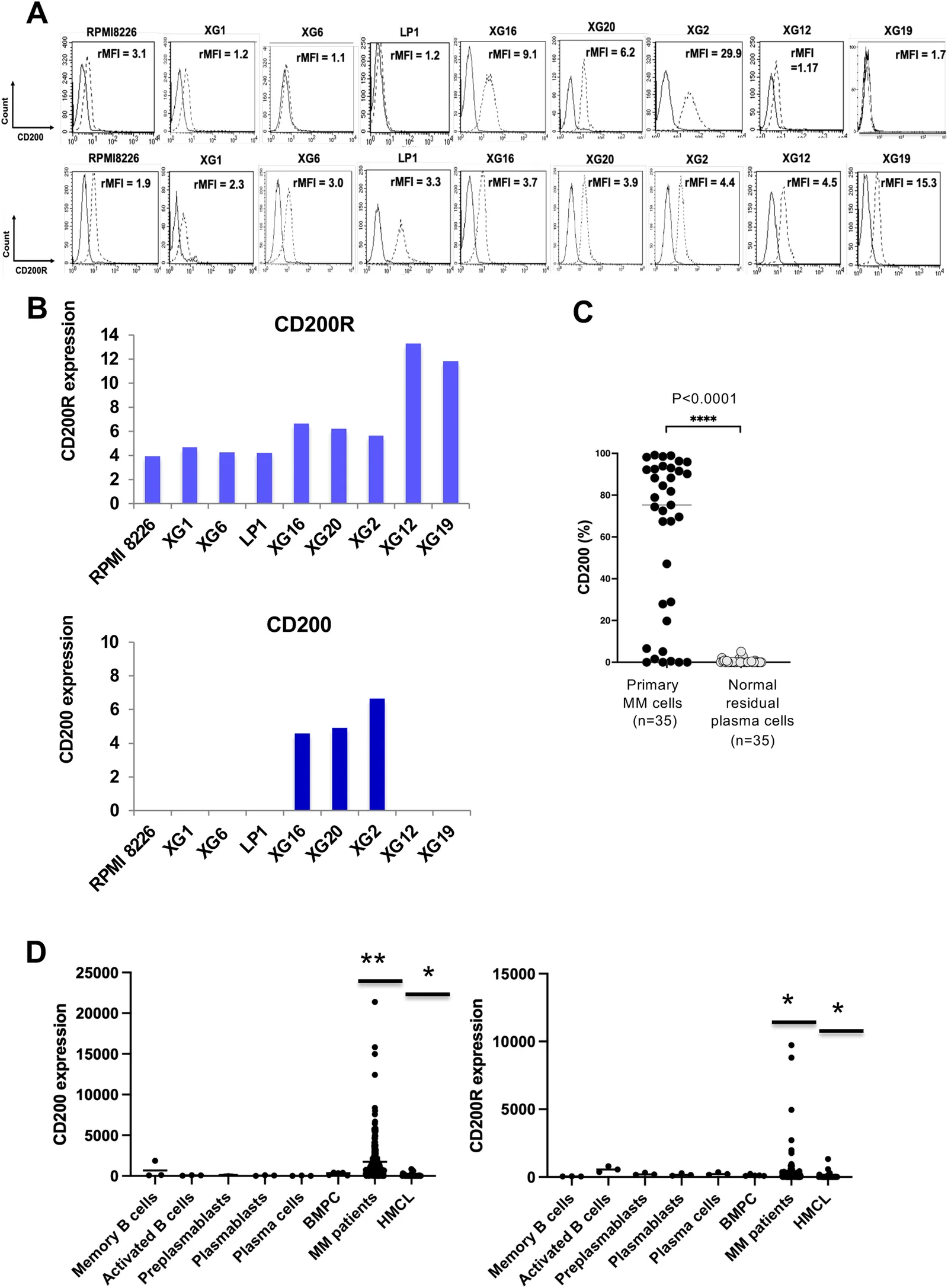
Expression of CD200 and of its receptor CD200R in normal and malignant plasma cells. (A) Cell surface expression of CD200 and CD200R was determined by flow cytometry using phycoerythrin (PE)-conjugated anti-CD200 (A) or (PE)-conjugated anti-CD200R MoAbs. Results are the mean fluorescence intensities (MFIs) obtained with the anti-CD200R1 or CD200 antibodies (dotted line). MFI of the isotype-matched control mAb is presented with solid line. (B) mRNA expression levels of CD200 and CD200R in nine HMCLs. Data are percentage of mRNA normalized by b2M expression. (C) CD200 protein expression on malignant MM cells and normal residual plasma cells was analyzed by flow cytometry using bone marrow from previously untreated MM patients (*N* = 35). The percentage of CD200 positive cells in malignant and normal plasma cells was analyzed. (D) CD200 gene expression was quantified using RNA-Sequencing in memory B cells (MBC), pre plasmablasts (PrePB), plasmablasts (PB), and plasma cells (PC) (*n* = 3), primary multiple myeloma (MM) samples (*n* = 207), and human myeloma cell lines (HMCLs) (*n* = 33). P values were calculated using a Wilcoxon test: *: *P* < 0.05 **: *P* < 0.01.

The expression of CD200 and of its receptor CD200R were first analyzed in 9 HMCLs using flow cytometry. CD200 was only expressed on the surface of three of them: XG16, XG20 and XG2 (the ratios of the MFI ranging from 1.1 to 29.9, Fig. 1**A**), whereas CD200R was present on the surface of all of these cell lines (the ratios of the MFI ranging from 1.9 to 15.3, Fig. 1**A**). These results were validated by real time-quantitative polymerase chain reaction (Fig. 1**B**).

Fresh bone marrow samples obtained from 35 newly diagnosed MM patients were then analyzed by MFC, to determine the level of expression of CD200 at the surface of primary MM cells (MMCs) and of residual normal plasma cells. As previously reported [11], CD200 was expressed at the surface of MMCs in 74,3% of the cases. In contrast, CD200 was absent at the surface of normal plasma cells (*P* < 0.0001, Fig. 1**C**) [36].

Using RNAseq data, *CD200* and *CD200R* gene expression levels were also evaluated, by RNAseq in normal memory B cells, normal activated B cells, normal preplasmablasts, normal plasmablasts, normal plasma cells generated *in vitro* [37], purified normal bone marrow plasma cells (*n* = 5), purified MMCs obtained from newly-diagnosed patients (*n* = 207) and HMCLs (*n* = 33) (Fig. 1**D**). *CD200* was significantly overexpressed in primary MMCs compared to normal cells, which did not express this gene (*P* < 0.05), and compared to HMCLs (*P* < 0.001). Interestingly, *CD200R* was overexpressed in normal MMCs (*P* < 0.05) compared to normal plasma cells. *CD200R* expression was heterogeneous in HMCLs with a lower expression compared to primary MMCs (*P* < 0.05).

Altogether, these data indicated that CD200 was not expressed by normal plasma cells, heterogeneously expressed at the surface of HMCLs and overexpressed in primary MMCs. CD200R was overexpressed in MMCs in comparison with normal plasma cells and HMCLs. All of the HMCLs expressed CD200R.

### Overexpression of CD200 promotes HMCLs growth in serum-free culture medium

To explore the biological function of CD200 in MM, LP1 and XG1, two CD200^-^ HMCLs, were transduced with a lentivirus containing a CD200 cDNA or a GFP cDNA as control. Three days after transduction, CD200 expression was detected by flow cytometry at the surface of 80 to 98% of the transduced cells, at a similar level of CD200 cell surface expression on primary MMCs (**supplementary Figures S1A,B,C**).

**Fig. 2:**
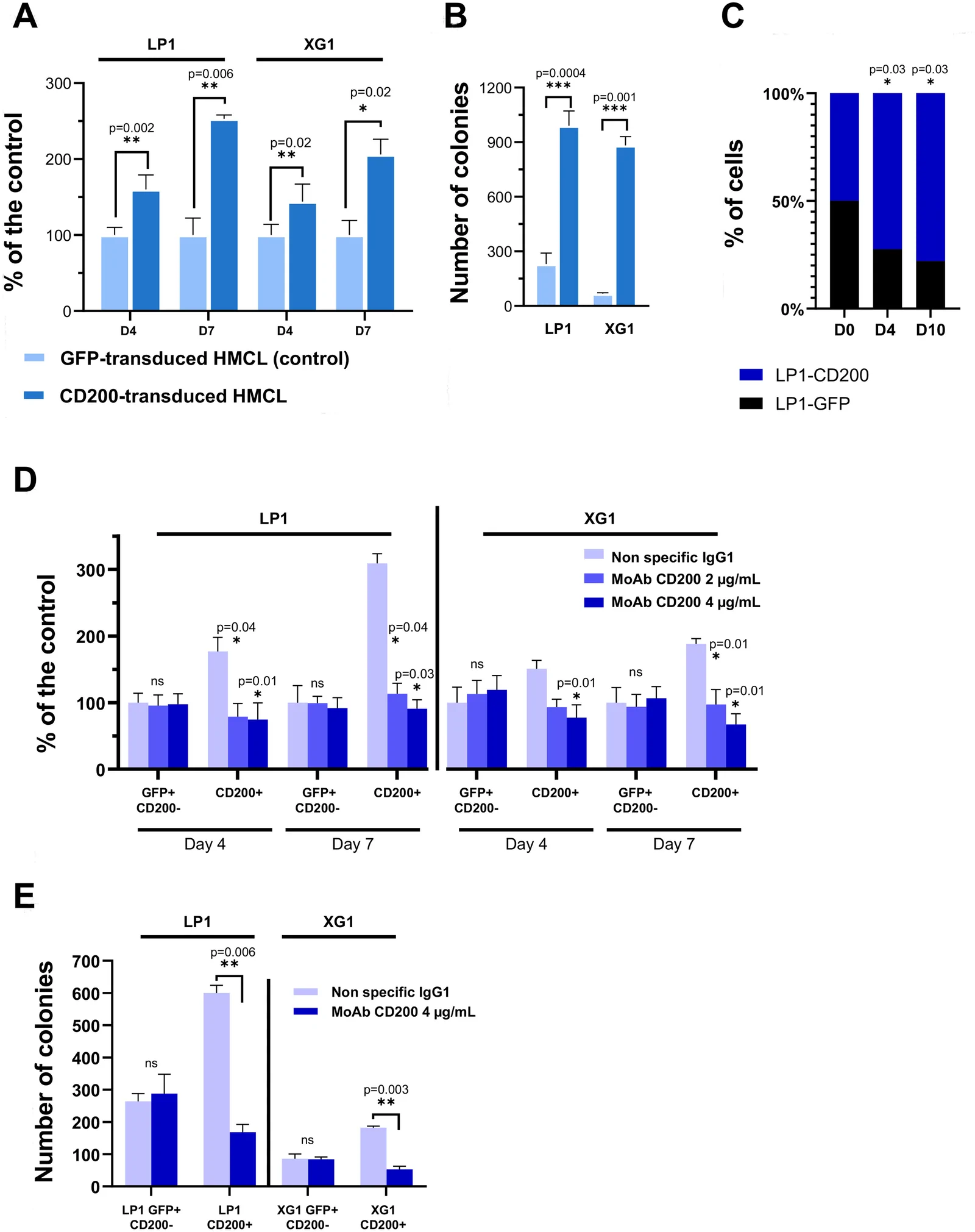
CD200 expression confers a proliferative advantage of MM cells in serum-free culture medium. Transfected HMCLs (LP1GFP, LP1CD200, XG1GFP and XG1CD200) were starved for 2 h and cultured during 7 days or 10 days without cytokine in SYNH serum-free medium (10^5^ cells/mL). (A) Results are the mean percentage (± SD) of the luminescent signal of CD200 ^positive^ cell lines compared with the GFP ^positive^ control cell lines determined in 3 separate experiments of six replicate culture wells. (B) Clonogenic assays were performed with a collagen semi solid culture medium without cytokines (Stem Alpha III). Ten thousand HMCLs were plated into a 35 mm tissue culture plate for layer agar cultures and cultured for 21 days. Results are the number of counted colonies (± SD) in each plate of 3 independent experiments. Picture of one representative experiment were presented. (C) Co-culture assay of LP1 GFP and LP1 CD200^positive^ cell lines was developed to investigate growth advantage of CD200^positive^ compared to CD200^negative^ MM cells. The percentage of the different populations was investigated using flow cytometry at day 0, 7 and 10. The mean percentage of three independent experiments was presented. (D) Transfected HMCLs (LP1GFP, LP1CD200, XG1GFP and XG1CD200) were starved for 2 h and cultured for 7 days without cytokine in SYNH serum-free medium in presence or absence of a neutralizing anti-CD200 MoAb (2µg/mL or 4µg/mL) or with control MoAb (4µg/mL). Results are the mean percentage (± SD – three independent experiments) of the luminescent signal of cell lines compared with that of the GFP^positive^ control cultures with the control MoAb. (D) Clonogenic assays were performed as previously described with or without a control MoAb (4µg/mL) or anti-CD200 MoAb (4µg/mL). Antibodies are added twice a week. Results represent the number of counted colonies (± SD) in each plate (3 independent experiments). *P* values were calculated using a Wilcoxon test for pairs ((*: *P* < 0.05; **: *P* < 0.01; NS: not significant).

In comparison with the control condition, CD200 expression significantly increased the growth of LP1 and XG1 HMCLs in SYNH serum-free culture medium (cell growth increase: 2.5 and 2.1-fold at day 7, *P* = 0.006 and *P* = 0.02, respectively for LP1 and XG1, Fig. 2**A** and **supplementary Figure S1D**).

In addition, after 21 days of culture in serum-free semi-solid culture medium, clonogenic assay demonstrated a significant higher number of colonies for CD200^+^ LP1 and CD200^+^ XG1 cells in comparison with the control condition (*P* = 0.0004 and *P* = 0.001, respectively for LP1 and XG1, Fig. 2**B and supplementary Figure S2E**).

Furthermore, in a culture of an initial mix of CD200^+^ LP1 cells and CD200^-^ LP1 cells, at a 1/1 ratio, a progressive disappearance of CD200^-^ cells was observed underlining a significant growth advantage provided by CD200 overexpression (*P* = 0.02 at day 10, Fig. 2**C**).

### Monoclonal antibody targeting CD200 inhibits myeloma cell growth induced by CD200 overexpression

To precise the role of CD200 on myeloma cell growth, a mouse IgG1 monoclonal antibody (MoAb) targeting CD200 (anti-CD200) was added in the culture medium.

The growth of CD200^+^LP1 cells treated with the anti-CD200 MoAb was significantly decreased (1.6-fold) at day 4 and day 7 (1.9-fold) compared to treatment with a non-specific anti-IgG1 MoAb (Fig. 2**D**). A similar result was obtained for CD200^+^XG1 HMCL, with a 2.3- and 4-fold decrease after 4 and 7 days of culture. Anti-CD200 MoAb resulted in a significant inhibition of CD200-mediated cell growth in MM cells (Fig. 2**D**).

These results were confirmed using a clonogenic assay. The anti-CD200 MoAb was used at a concentration of 4 µg/mL and added twice a week during 21 days. No inhibitory effect on colony formation was identified for CD200^-^ LP1 and XG1 HMCLs in the presence of anti-CD200 MoAb. In contrast, the growth of CD200^+^ LP1 and XG1 cells was significantly impaired in the presence of the anti-CD200 MoAb (Fig. 2**E**, *P* = 0.006 and *P* = 0.003, respectively, **supplementary Figure S2A**).

### CD200R is not involved in myeloma cell growth induced by CD200

**Fig. 3:**
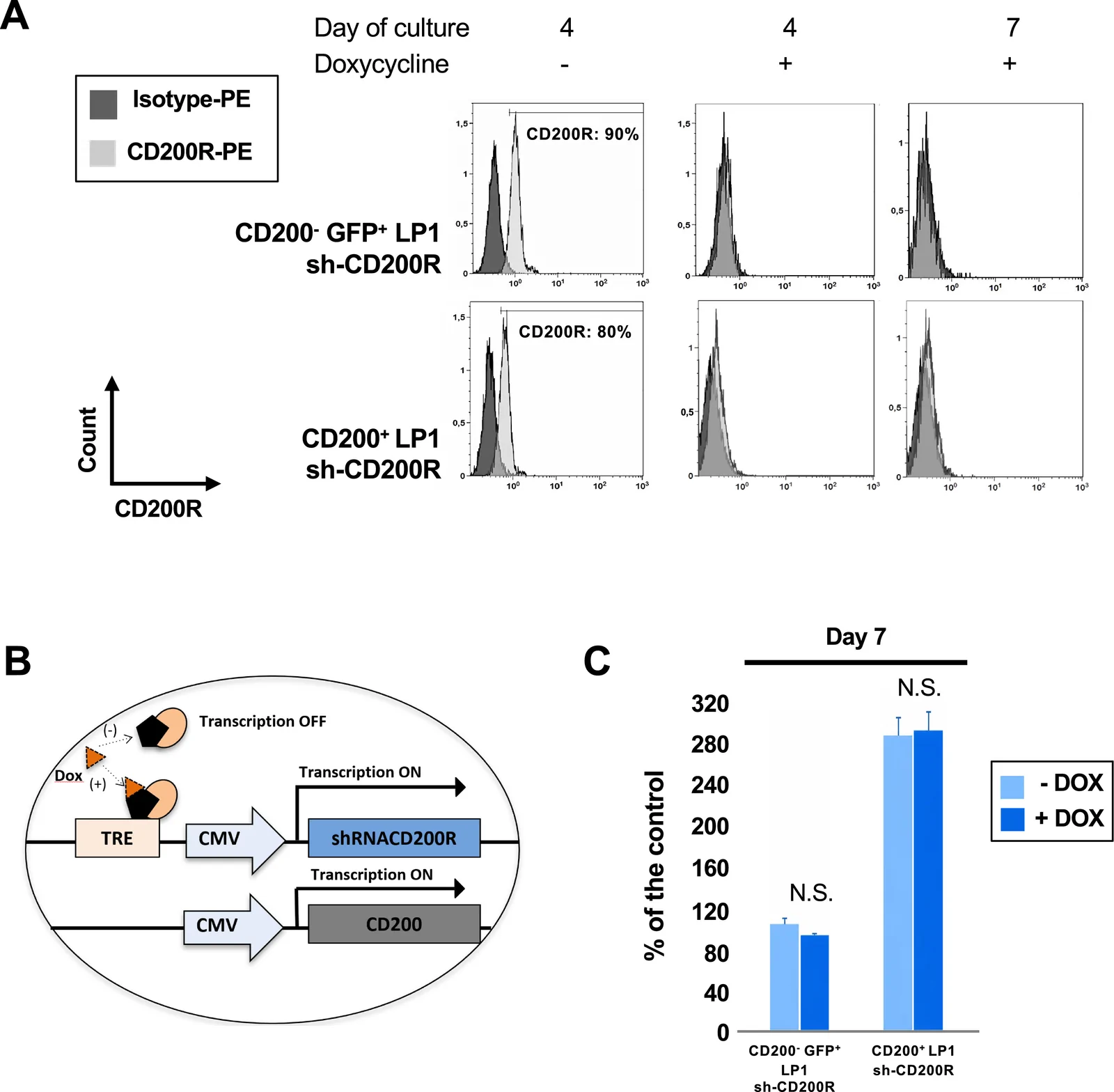
The growth advantage of CD200^positive^ MM cells is not affected by the CD200R depletion. (A) The effects of inducible CD200R depletion in LP1CD200^negative^ cell line or in LP1CD200^positive^ cell line was investigated with flow cytometry. (B) Representation of the lentiviral approaches used for inducible CD200R depletion as previsouly described [30] in a context of constitutive CD200 overexpression. (C) The effects of inducible depletion of CD200R (+ Doxycycline) in LP1 CD200 ^negative^ cell line or in LP1 CD200 ^positive^ cell line were investigated with Cell Titer Glo assay. Results are the mean percentages (± SD) of the luminescent signal of each group compared to the control (LP1_shCD200R without doxycycline) in 3 independent experiments including sixplicate culture wells. *P* values were calculated using a Wilcoxon test for pairs. NS: not significant.

To investigate the role of CD200R in myeloma cell growth induced by CD200, CD200^-^ GFP^+^ LP1 cells and CD200^+^ LP1 cells were transduced with a lentivirus containing the tetracycline repressor (TR). HMCLs were then transduced with a lentiviral vector containing a shRNA targeting CD200R under the control of the Tet operator (Fig. 3).

Doxycycline treatment resulted in a significant reduction of CD200R protein expression (95 to 100%) at day 4 and day 7 (Fig. 3**A**).

Interestingly, the absence of CD200R at the surface of CD200^-^ LP1 cells or CD200^+^LP1 cells didn’t affected MM cell growth in serum free culture medium (Fig. 3**C**). The same results were observed when the cells where cultured FCS supplemented culture medium (**supplementary Figure S3**).

Altogether, these results suggest that the induction of myeloma cell growth mediated by CD200 is not dependent on the presence of CD200R.

### Myeloma cell growth induced by CD200 depends on IGF1R

**Fig. 4:**
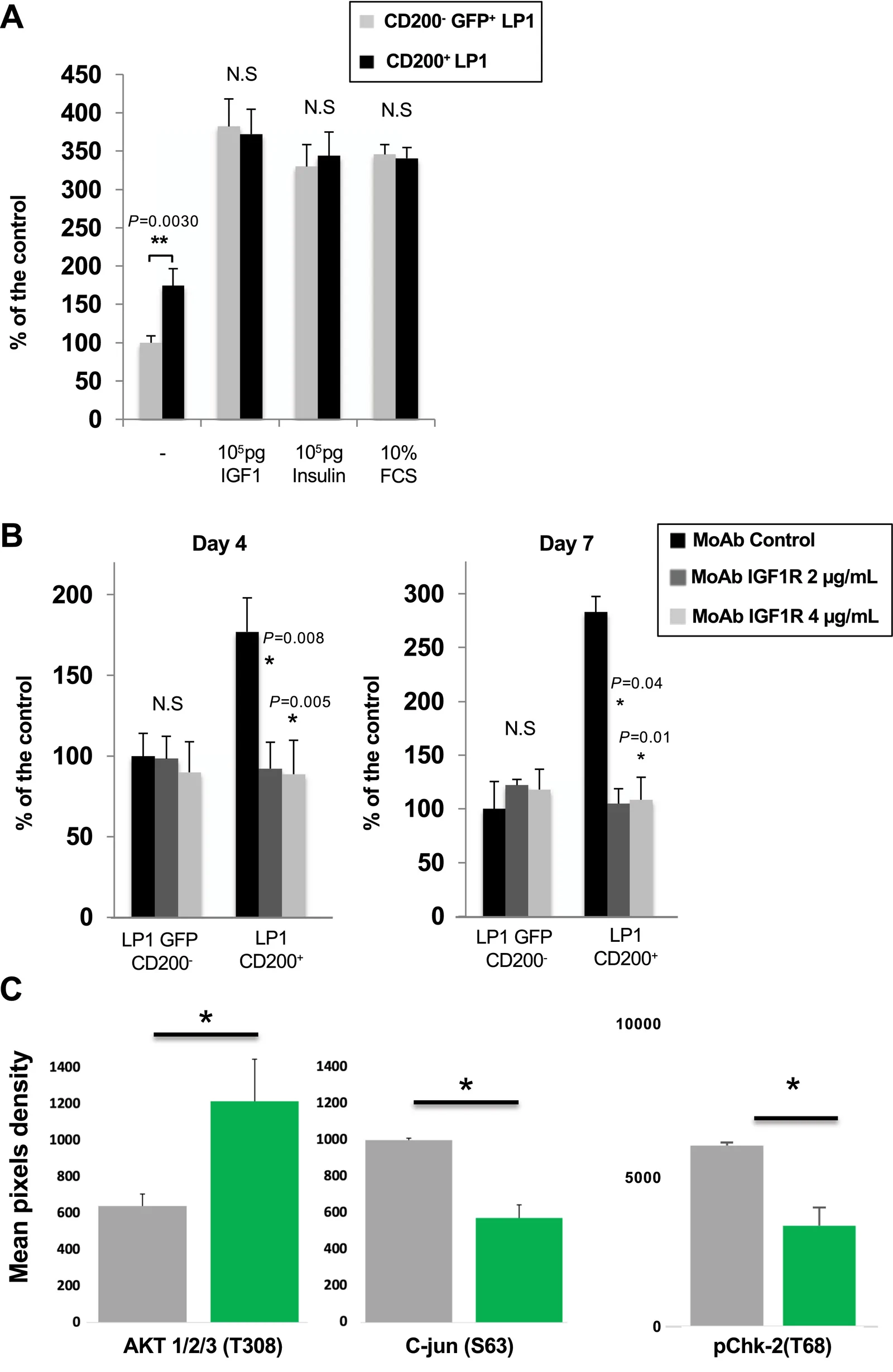
MM cell growth mediated by CD200 is dependent on an IGF-1/IGF-1R loop. (A) HMCLs were starved for 2 h in serum free culture medium and cultured for 4 days in SYNH serum free culture medium without growth factor (-) or with either IGF-1 (10^5^pg/mL), insulin (10^5^pg/mL) or 10% fetal calf serum (FCS). *P* values were calculated using a Wilcoxon test for pairs (**: *P* < 0.01). NS: not significant. (B) HMCLs were starved for 2 h in serum free culture medium and cultured for 4 or 7 days in serum free culture medium with a neutralizing anti-IGF-1R MoAb (2µg/mL or 4µg/mL) or a non-relevant control MoAb (4µg/mL). Results are the mean percentages (± SD) of the luminescent signal performed in 3 independent experiments with sixplicate culture wells. *P* values were calculated using a Wilcoxon test for pairs (**<0.01). NS: not significant. (C) Apoptosis and signaling pathways activated by CD200 expression in MM cells. Proteins accumulations were monitored after 4 days of culture in SYNH serum free culture medium using LP1 GFP control or LP1 CD200 HMCLs and proteome profiler arrays as previously described [63]. Relative amount was calculated as the mean of pixel density. *: *P* < 0.05.

Adding IGF1 or FCS in SYNH serum-free culture medium abolished the growth advantage provided by CD200 expression in LP1 cells. The same result was obtained following the addition of recombinant insulin, which is also a potent MM growth factor by activating a hybrid receptor composed of IGF1R and insulin R (INSR) [23] (Fig. 4**A** and **supplementary Figure S4**). These data suggest that CD200 could promote MM cell growth through an IGF1-IG1R axis [22].

Interestingly, the growth of XG12 IGF1R negative HMCL was not stimulated by the addition of IGF1 or insulin in the culture medium (**supplementary figure S5A-B**). Overexpression of CD200 in XG12 HMCL did not provided a cell growth advantage (**supplementary Figures 5B-C**) and did not increase the number of colonies in clonogenic assays (**supplementary Figure 5D**).

Of note, IGF1 production was measured in the supernatant of CD200^-^ GFP^+^ LP1 cells and CD200^+^ LP1 cells. No significant difference was found between CD200^+^ and CD200^-^ MM cells (**supplementary Figure S6A**). Treatment with an anti-IGF1R MoAb resulted in a selective abrogation of the CD200-mediated proliferative activity in CD200^+^ HMCLs (Fig. 4**B**). These results demonstrated that the induction of myeloma cell growth by CD200 is dependent on IGF1R.

**Fig. 5:**
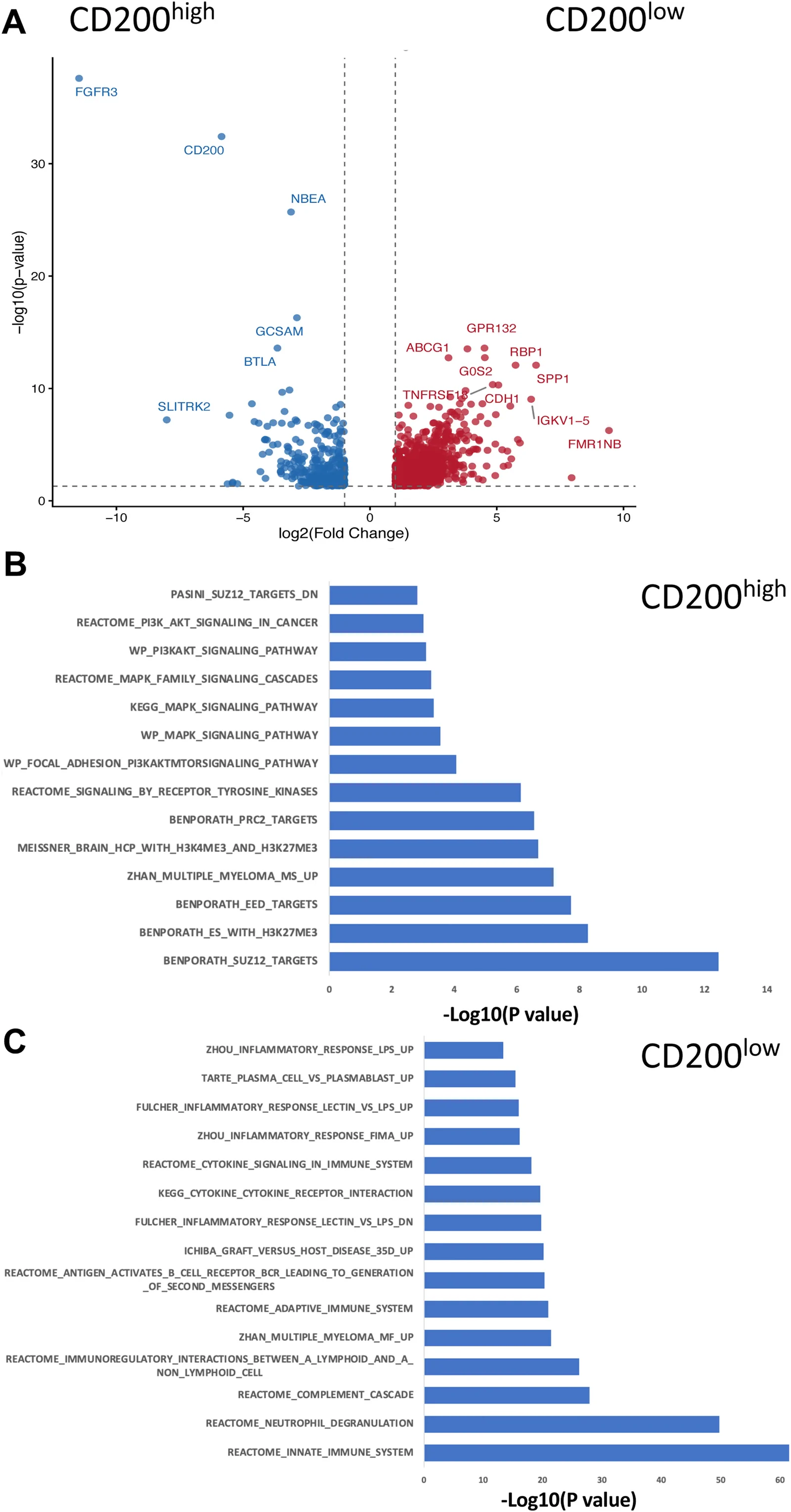
CD200^high^ MM cells from patients are associated with a significant enrichment in PI3K and MAPK signaling pathways. (A) Using RNA sequencing data from purified MM cells from patients (*n* = 208), we compared the genes differentially expressed between MM patients with high or low CD200 expression (Mean *CD200* expression ± SD). Using DESEQ2 R package, 1246 genes were identified to be differentially expressed between CD200^high^ and CD200^low^ MM cells (Fold change ≥ 2, FDR < 0.05). (B) GSEA analysis performed on genes significantly differentially expressed between CD200^low^ and CD200^high^ MM patients (ratio ≥ 2, FDR < 0.05). Only the significant signaling pathways are represented (FRD q ≤ 0,05) as log10 (*p* value). (B) Pathways significantly enriched in MM cells from CD200^high^ patients (353 genes). (C) Pathways significantly enriched in MM cells from CD200^low^ patients (894 genes).

Using RNA sequencing data from purified MM cells from patients (*n* = 208), we compared the genes differentially expressed between MM patients with high or low CD200 expression (Mean *CD200* expression ± SD). Using DESEQ2 R package, 1246 genes were identified to be differentially expressed between CD200^high^ and CD200^low^ MM cells (Fold change ≥ 2, FDR < 0.05) (**Supplementary Table S1**). Among the 353 genes significantly upregulated in CD200^high^ MM cells (Fig. 5**A**), GSEA analysis revealed a significant enrichment of Polycomb repressive complex (PRC2) target genes and genes involved in PI3K/AKT signaling, and MAPK signaling (Fig. 5**B**). MM cells from CD200^low^ patients presented a significant enrichment in inflammatory response genes, immune activation genes and cell to cell communication (Fig. 5**C**). IGF1R activation by IGF1 results in PI3K activation supporting MM cell proliferation and survival [22,23,38]. These results support the interplay between CD200 and IGF1R to support MM cell growth.

Using a proteome array assay, we investigated the pathways involved in apoptosis and cell cycle comparing LP1 GFP and LP1 CD200 MM cell lines. Of interest, we identified a significant induction of T308 AKT phosphorylation in CD200^+^ LP1 compared to the control. It was associated with a decrease of S63 phosphorylation of c-Jun together with a decrease in T68 Chk2 phosphorylation in LP1 CD200 compared to the control (Fig. 4**C**). No significant differences in the protein levels of pro and anti-apoptotic proteins were identified (**Supplementary Figure S7**). Altogether, our data support a role of CD200 as a MM growth factor through IGF1R signaling and PI3K activation.

### Prognostic value of CD200 and IG1R expression in MM

**Fig. 6:**
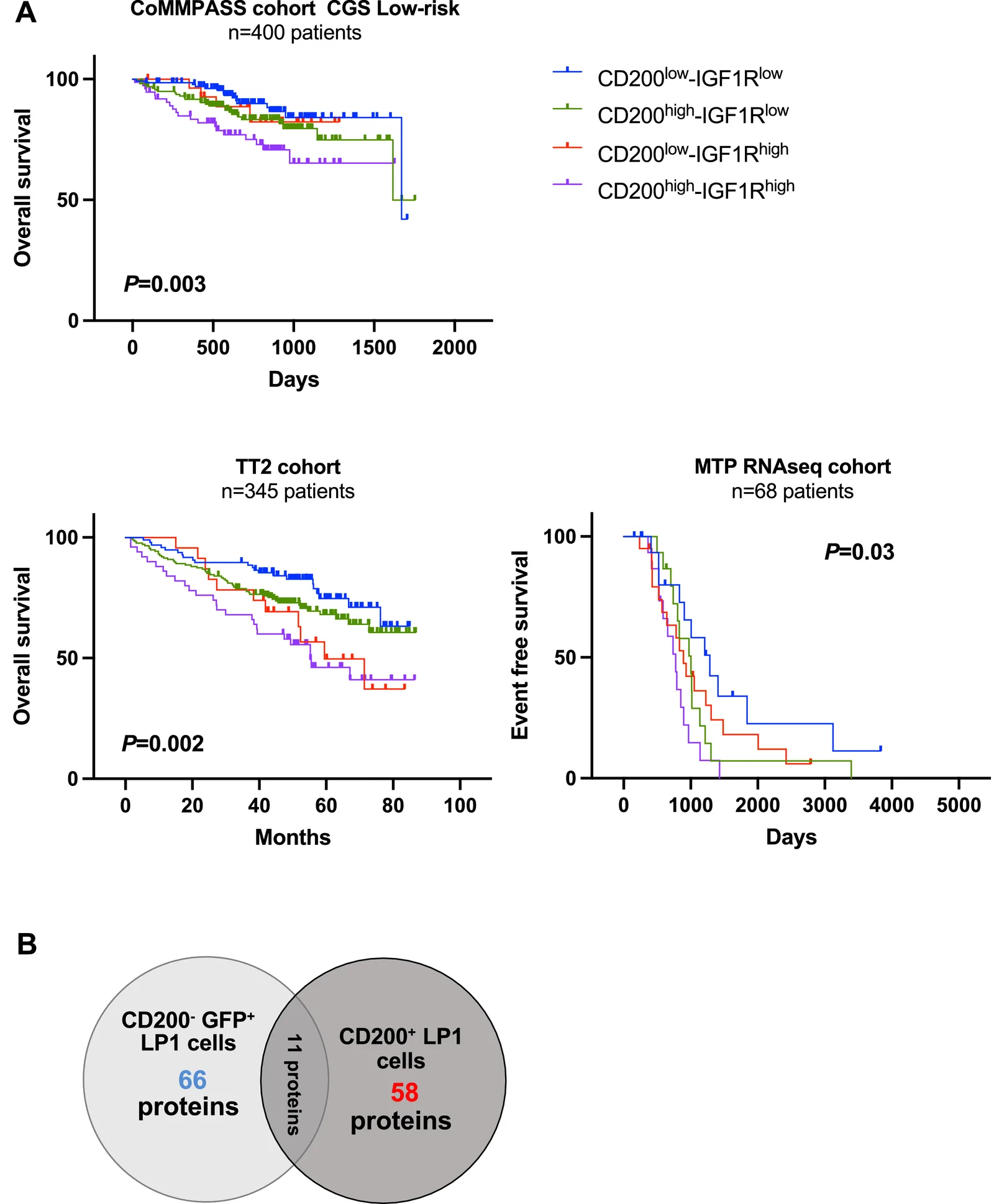
Prognostic value of *IGF-1R* and *CD200* gene expression in multiple myeloma patients and protein interaction. (A) Kaplan-Meier curves showing the overall survival (OS) of patients from the publicly available CoMMpass cohort based on their IGF1R and CD200 expression levels. The analysis was completed in the MM patients from the CoMMPASS cohort characterized by a low risk according to the Consensus Genomic Staging (CGS, International myeloma society/International myeloma working group) (*n* = 400). Maxstat R algorithm revealed that concomitant high expression of CD200 and IGF1R is associated with a significant shorter event overall survival (OS). *CD200* and *IGF-1R* gene expression were investigated in purified myeloma cells from patients using Affymetrix U133P microarrays from the publicly available TT2 cohort (Arkansas (GEO accession number GSE2658)). KM curves have been built using Prism software. These data were validated using another independent cohort of newly diagnosed MM patients treated by high dose chemotherapy and autologous transplantation (Montpellier RNAseq cohort) [64]. RNAseq data of purified MM cells were analyzed using the same methodology. *P* value was defined using Logrank test. (B) LP1 GFP and LP1 CD200 cells were starved and culture in SYNH serum free culture medium for 4 days. IGF1R immunoprecipitation (IP) was performed with an anti-IGF-1R antibody or with a non-relevant control antibody and analyzed by mass spectrometry as previously reported [30]. The selection of protein only immunoprecipitated with IGF-1R in LP1 GFP cells compared to control antibody revealed 77 proteins. IGF1R interactome revealed 58 proteins including CD200 specifically in LP1 CD200 cells.

The international myeloma society/International myeloma working group provided recommendations to define high-risk MM patients [39]. However, functional high-risk MM were reported as an unexpected early relapse of disease in the absence of baseline molecular or clinical risk factors [40]. Using the publicly available CoMMPASS cohort, we investigated the prognostic value of CD200 and IGF1R expression in low-risk patients defined by the Consensus Genomic Staging (CGS) [39]. Of interest, high *IGF1R* and *CD200* gene expression was associated with a significant shorter overall survival in the low-risk MM patients defined by the CGS (Fig. 6**A**). We also analyzed a cohort of newly diagnosed MM patients treated by high-dose chemotherapy and autologous stem cell transplantation (ASCT) (TT2 cohort of 345 patients). MM patients with high *IGF1R* expression or high *IGF1R* and *CD200* gene expression are characterized by a decreased overall survival (Fig. 6**A**). We validated these results using an independent cohort of newly diagnosed MM patients with RNA sequencing characterization of purified MM cells (*n* = 68) [41]. Concomitant high expression of CD200 and IGF1R was also associated with a significantly shorter event free survival (Fig. 6**A**).

### Co-IP/MS identifies CD200 in the IGF1R-associated protein complex and supports an association between CD200 and IGF1R

Since no anti-CD200 MoAb was developed for immunoprecipation experiments, IGF1R was immunoprecipitated in non-denaturing conditions using CD200^-^ GFP^+^ and CD200^+^ LP1 HMCLs. After immunoprecipitation, IGF1R was detected by western blot (**supplementary Figure S6B**) and CD200 was detected in the immunoprecipitated fraction of CD200^+^ LP1 cells (**supplementary Figure S6C**). These results supported a direct or indirect association between CD200 and IGF1R at a protein level.

The IGF1R-associated protein complex was analyzed by mass spectrometry as reported [30](Fig. 6**B**). 87 proteins were identified using the fraction corresponding to CD200^-^ GFP^+^ LP1 (Supplementary Table S2), whereas 58 proteins, including CD200, were specifically detected only in the fraction from CD200^+^ LP1 cells (Table 1). Using reactome analysis, we identified a significant enrichment in proteins involved in post-translational protein phosphorylation, translation, immune response and hedgehog pathway (**Supplementary Table S3**). No overlap was identified between the pathways significantly enriched in the RNAseq signatures characterizing the CD200^high^ MM cells and the pathways enriched in the IGF1R interacting proteins. Altogether, our data identified CD200 in the IGF1R-associated protein complex and supports an association between CD200 and IGF1R to promote MM cell growth.

**Table 1:** IGF1R was immunoprecipitated in the LP1 GFP control and LP1 CD200+ cell lines and analyzed by mass spectrometry. Anti-normal IgG antibody was used as a negative control. The list presents the protein specifically associated with IGF1R in the LP1 CD200+ cell line and not in the LP1 GFP control cell line. The data include only the proteins that are not identified in the Ig control.

Protein name	Description	Meta Score B
1A33_HUMAN	major histocompatibility complex, class I, A	129.9
ANGT_HUMAN	angiotensinogen (serpin peptidase inhibitor, clade A, member 8)	86.8
ANM1_HUMAN	protein arginine methyltransferase 1	79
ANT3_HUMAN	serpin peptidase inhibitor, clade C (antithrombin), member 1	32.1
ANXA7_HUMAN	annexin A7	31.6
APOA4_HUMAN	apolipoprotein A-IV	109.7
ARP2_HUMAN	ARP2 actin-related protein 2 homolog (yeast)	32.8
BASI_HUMAN	basigin (Ok blood group)	50.8
CO4B_HUMAN	complement component 4B (Chido blood group)	68.1
CTF18_HUMAN	CTF18, chromosome transmission fidelity factor 18 homolog (S. cerevisiae)	68.8
DAPK1_HUMAN	death-associated protein kinase 1	94.6
EDC4_HUMAN	enhancer of mRNA decapping 4	54.5
EIF3M_HUMAN	eukaryotic translation initiation factor 3, subunit M	82.1
ENPL_HUMAN	heat shock protein 90 kDa beta (Grp94), member 1	33
GEMI5_HUMAN	*gem* (nuclear organelle) associated protein 5	35
GNA13_HUMAN	guanine nucleotide binding protein (G protein), alpha 13	61.4
HNRL1_HUMAN	heterogeneous nuclear ribonucleoprotein U-like 1	32.4
HS90A_HUMAN	heat shock protein 90 kDa alpha (cytosolic), class A member 1	113.4
HS90B_HUMAN	heat shock protein 90 kDa alpha (cytosolic), class B member 1	183.5
IF2G_HUMAN	eukaryotic translation initiation factor 2, subunit 3 gamma, 52kDa	62.5
IGHG2_HUMAN	immunoglobulin heavy constant gamma 2 (G2m marker)	324.4
IGHG4_HUMAN	immunoglobulin heavy constant gamma 4 (G4m marker)	571.6
IMDH2_HUMAN	IMP (inosine 5′-monophosphate) dehydrogenase 2	37.1
LDHB_HUMAN	lactate dehydrogenase B	64.5
MCM2_HUMAN	minichromosome maintenance complex component 2	40.3
MCM4_HUMAN	minichromosome maintenance complex component 4	75.1
MDHM_HUMAN	malate dehydrogenase 2, NAD (mitochondrial)	36.1
OX2G_HUMAN	CD200	52.4
PDCD4_HUMAN	programmed cell death 4 (neoplastic transformation inhibitor)	79.3
PDIA1_HUMAN	prolyl 4-hydroxylase, beta polypeptide	56.9
PLSI_HUMAN	plastin 1	38.5
POTEJ_HUMAN	POTE ankyrin domain family member J	143.8
PPOX_HUMAN	protoporphyrinogen oxidase	40.7
PRP4_HUMAN	PRP4 pre-mRNA processing factor 4 homolog (yeast)	31.8
PRS6A_HUMAN	proteasome (prosome, macropain) 26S subunit, ATPase, 3	57.4
PSAL_HUMAN	Puromycin-sensitive aminopeptidase-like protein	61.9
PSD11_HUMAN	proteasome (prosome, macropain) 26S subunit, non-ATPase, 11	86.4
RAN_HUMAN	RAN, member RAS oncogene family	53.2
RCN1_HUMAN	reticulocalbin 1, EF-hand calcium binding domain	37
RFC2_HUMAN	replication factor C (activator 1) 2, 40kDa	47.5
RLA0_HUMAN	ribosomal protein, large, P0	68.4
RS16_HUMAN	ribosomal protein S16	37.4
RS8_HUMAN	ribosomal protein S8	35.7
RT27_HUMAN	mitochondrial ribosomal protein S27	49
STAT2_HUMAN	signal transducer and activator of transcription 2, 113kDa	50.9
SYAM_HUMAN	alanyl-tRNA synthetase 2, mitochondrial (putative)	35
SYDM_HUMAN	aspartyl-tRNA synthetase 2, mitochondrial	37.7
SYMC_HUMAN	methionyl-tRNA synthetase	55.1
TCPH_HUMAN	chaperonin containing TCP1, subunit 7 (eta)	30.3
TERA_HUMAN	valosin containing protein	82.4
TMOD3_HUMAN	tropomodulin 3 (ubiquitous)	78.9
TPIS_HUMAN	triosephosphate isomerase 1	73.6
TRFE_HUMAN	transferrin	53.9
TRUA_HUMAN	pseudouridylate synthase 1	41.6
U2AF2_HUMAN	U2 small nuclear RNA auxiliary factor 2	43.6
VTDB_HUMAN	group-specific component (vitamin D binding protein)	30.7
XRCC5_HUMAN	X-ray repair complementing defective repair	30.7

## Discussion

In this study, we have shown that CD200 acts as a myeloma growth factor through interaction with IGF1R. CD200 is expressed in 70% of MM patients and IGF1/IGF1R signaling is known to be a major myeloma growth factor [22,23]. IGF1 binds to IGF1R expressed on plasma cells. It results in PI3K activation through the phosphorylation of insulin receptor substrate 1 and 2 (IRS1 and 2) and tyrosine residues. When activated, PI3K recruits PDK1 and AKT to the cell surface followed by AKT phosphorylation at Threonine 308 and activation. This activation induces MM cell proliferation and survival. However, activating mutations of AKT and PI3K have not been reported in MM [42]. Our group previously reported that IGF1 and IL-6 are the major MM growth factor and that IL-6, HGF or HB-EGF-mediated survival of MM cells is dependent on the presence an autocrine IGF1-IGF1R loop [22]. Gene-expression profiling across normal PCs, MGUS, smoldering MM and MM cells defined IGF1R overexpression enriched in poor-prognostic subtypes [43,44]. Significant decreased proliferation was observed upon the CRISPR-Cas9 knockout of IGF1R in several MM cell lines, supporting the observation that MM depends on IGF1R [44]. IGF-I signaling is implicated in resistance to standard-of-care MM drugs including proteasome inhibitors, IMiDs, and corticosteroids and IGF-1R targeting can reverse this resistance in preclinical models [45]. While IGF1R is widely considered as a key oncogenic driver in MM with encouraging results in preclinical studies, these findings have not translated into meaningful clinical responses when IGF1R inhibitors are used as monotherapy [46]. It could be related to compensatory signaling through alternative growth factor pathways, potentially facilitated by IGF1R's capacity to form hybrid receptors [47]. IGF1R could form hybrid receptors with other receptor tyrosine kinases including INSR, EGFR and HER2 [48,49]. IGF1R could also interact with integrins and cadherins [50].

MM cells expressing CD200 show a significant growth advantage in serum-free medium and clonogenic assays. This effect is abrogated by anti-CD200 and anti-IGF1R antibodies. The IGF1R dependent MM cell growth activity of CD200 is emphasized by the lack of effect on the IGF1R negative XG12 HMCL. Furthermore, we could demonstrate that these effects are independent of CD200 receptor since inducible depletion of CD200R didn’t affect the MM cell growth advantage mediated by CD200 expression. Here, we identified CD200 in the IGF1R-associated protein complex in MM cells associated with AKT phosphorylation at Threonine 308 and activation. These results are supported by a significant enrichment of genes related to PI3K and MAPK signaling pathway among the genes significantly overexpressed in purified MM cells from CD200^high^ patients (**supplementary Figure S7**). AKT phosphotylation in CD200^+^ MM cells was associated with a decrease of S63 phosphorylation of c-Jun together with a decrease in T68 Chk2 phosphorylation compared to the control. In breast cancer, increased PI3K pathway activity was reported alongside suppression or downregulation of the CHEK2 pathway, suggesting a functional inverse relationship in tumor progression [51]. CD200 expression has been shown to be associated with a poor outcome in MM patients treated by high dose chemotherapy and autologous stem cell transplantation [11] or more recently by anti-CD38 monoclonal antibodies [41]. The poor prognosis associated with CD200 expression in MM was related to its immunosuppressive biological functions. CD200 expression is associated with regulatory T cell expansion [16] and inhibition of antitumoral functions of NK cells [41,52]. However, the immune functions of CD200 are mediated by the interaction with the CD200R expressed by immune cells [53]. Targeting of CD200-CD200R axis was shown to improve the MM cell lysis mediated by anti-CD38 MoAb [41]. Furthermore, the CD200-CD200R axis has been shown to enhance CAR-T cell efficacy [19]. The structure of CD200 was previously solved in complex with CD200R [54]. CD200 features an N-terminal Ig V-like domain followed by a smaller C2-like domain. CD200 exhibits a topology including a short α-helix in the EF loop. Its C2-like domain shows exceptionally high B-factors. Beyond the conserved disulfide bond between β-strands B and F in both IgSF domains, the V-like domain features an exposed disulfide between β-strands F and G [54]. It appears of major interest to develop experiments with different domain truncations and random mutagenesis of CD200 to define the domains involved in the interaction with IGF1R. IGF1R and MET were shown to associate with the detergent-resistant regions of the plasma membrane [55,56], enriched with several types of sphingolipids and cholesterol, enabling receptor clustering, which is essential for their complete activation [57,58]. In this context, in silico predictions models may be of major interest. The advent of AlphaFold has revolutionized structural biology and may help the identification of the domains involved in direct IGF1R and CD200 interactions or identify bridging proteins that could support indirect association [59]. Furthermore, our results support future investigations to define how CD200 may affect IGF1R dimerization, internalization or ligand binding affinity in MM.

High IGF1R expression was previously reported to be associated with an adverse outcome in MM patients [22]. Furthermore, concomitant high expression of *CD200* and *IGFR1* is associated with an adverse outcome. Samalizumab, a humanized anti-CD200 monoclonal antibody, is currently in development [53]. It functions primarily by blocking the CD200–CD200R interaction, thereby indirectly restoring lymphocyte-mediated immune activity. The most common drug-related grade 3 - 4 toxicities were blood and lymphatic system disorders (anemia, neutropenia, and thrombocytopenia) reported in 12% of the patients [53]. IGF1R has long been viewed as promising target in newly diagnosed and relapsed/refractory MM patients, as well as other cancers. However, no IGF1R inhibitors have been approved for cancer treatment to date [60]. Anti-IGF1R antibody CP-751,871 was safe and well tolerated in patients with MM in a phase I study [61]. However, it will be of major interest to investigate how these antibodies could affect the interaction between CD200 and IGF1R. More recently, Han Z et al. reported that ACOX1 inhibition affects lipid-protein interactions and the activation of IGF1R in MM cells [62]. Mechanistically, inhibiting ACOX1 changed how MET and IGF1R interact with a cerebroside analog, and selectively reduced the association of these kinases with plasma membrane signaling platforms. This approach in association with CD200 targeting may provide new therapeutic avenues to target CD200 mediated cell growth myeloma function and its immunosuppressive functions associated with resistance to immune based therapies.

## Funding

The J. Moreaux research group was supported by grants from INCA PLBIO22 PIC-ASO (INCA_16734), ANR-23-CE15-0016-01 EPI-B-PLASMADIFF, SIRIC Montpellier Cancer (INCa-DGOS-INSERM- ITMO Cancer_ 18004), 10.13039/100026947ARC foundation PGA EpiMM3D, FFRMG (AAP-FFRMG-2021), AAP ECOPHYTO – PELYCANO (This action is led by the Ministries for Agriculture and Food Sovereignty, for an Ecological Transition and Technical Cohesion, for Health and Prevention, and for Higher Education and Research, with the financial support of the French Office for Biodiversity, as part of the call for projects on the Ecophyto II+ plan "Pgytosanitary products: from exposure to impacts on human health and ecosystems towards an integrated "one health" approach", with the fees for diffuse pollution coming from the Ecophyto II+ plan), MSDAVENIR EpiMuM-3D, Institut Universitaire de France and by the European Union (Project 101097094 — ELMUMY. Views and opinions expressed are however those of the author(s) only and do not necessarily reflect those of the European Union or HADEA. Neither the European Union nor the granting authority can be held responsible for them.). Views and opinions expressed are however those of the author(s) only and do not necessarily reflect those of the European Union or HADEA. Neither the European Union nor the granting authority can be held responsible for them.

## CRediT authorship contribution statement

**Angélique Bruyer:** Writing – original draft, Investigation, Formal analysis. **Nicolas Robert:** Investigation, Formal analysis. **Jules Varbedian:** Writing – original draft, Investigation, Formal analysis. **Elvira Garcia De Paco:** Writing – original draft, Investigation, Formal analysis. **Lauryn Jaffory:** Investigation, Formal analysis. **Stéphanie Boireau:** Investigation, Formal analysis. **Christophe Hirtz:** Writing – original draft, Methodology, Investigation, Formal analysis. **Jérôme Vialaret:** Writing – original draft, Methodology, Formal analysis. **Pierre Martineau:** Writing – original draft, Investigation, Formal analysis. **Bruno Robert:** Writing – original draft, Methodology, Investigation, Formal analysis. **Guilhem Requirand:** Investigation, Formal analysis. **Guillaume Cartron:** Writing – original draft, Investigation, Formal analysis. **Laure Vincent:** Writing – original draft, Investigation, Formal analysis. **Charles Herbaux:** Writing – original draft, Methodology, Investigation. **Jérôme Moreaux:** Writing – review & editing, Writing – original draft, Supervision, Methodology, Investigation, Funding acquisition, Conceptualization. **Caroline Bret:** Writing – review & editing, Writing – original draft, Supervision, Investigation, Formal analysis, Data curation, Conceptualization.

## Declaration of competing interest

The authors declare that they have no known competing financial interests or personal relationships that could have appeared to influence the work reported in this paper.

## Data Availability

For original data, please contact c-bret@chu-montpellier.fr and jerome.moreaux@igh.cnrs.fr. Gene expression data have been deposited in the ArrayExpress public database under accession numbers E-TABM-937, E-MTAB-372 and E-TABM-1088.

## Appendix Supplementary materials

Image, application 1

Image, application 2

Image, application 3

Image, application 4
