# Supplementary material for: CD200 is a potent new growth factor in Multiple Myeloma dependent on IGF1 receptor signaling
Source: Transl Oncol. 2026 Aug 28;72:103000. doi: 10.1016/j.tranon.2026.103000 (PMC13545282; doi:10.1016/j.tranon.2026.103000)
Supplement: Supplementary file 1 [file mmc1.pdf]

Figure S1

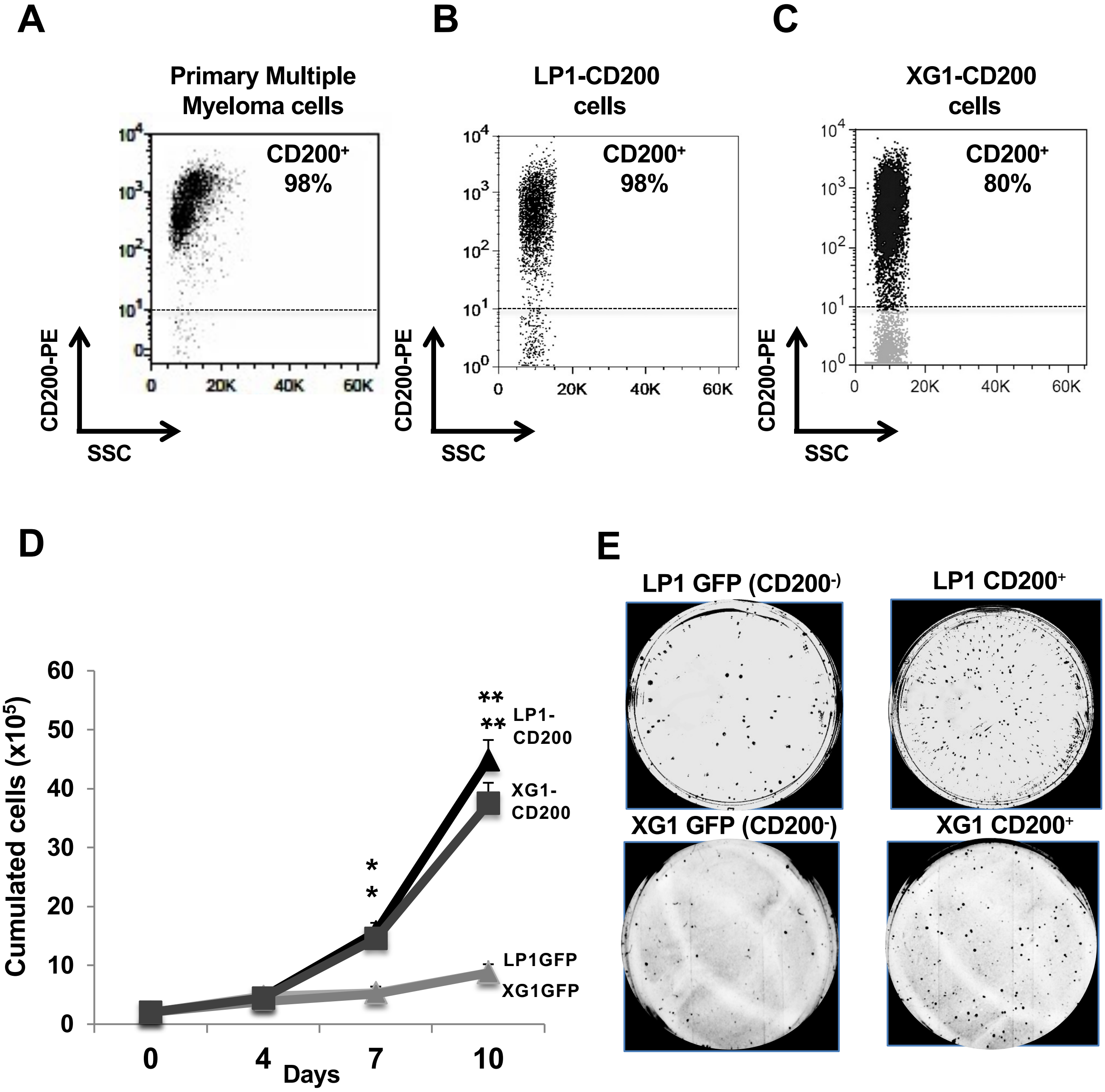

Supplementary Figure S1: (A,B,C) Cell surface expression of CD200 was determined by flow cytometry using phycoerythrin (PE)-on CD138+ MM cells from primary samples from patients and transduced LP1 and XG1 HMCLs. (D) LP1 and XG1 GFP or CD200 were starved for 2 hours in serum free culture medium and cultured for 10 days in SynH serum free culture medium. Cumulated cell growth was quantified. *P* values were calculated using a Wilcoxon test for pairs (**P* <0.05; ***P* <0.01). (E) Clonogenic assays were performed with a collagen semi solid culture medium without cytokines (Stem Alpha III). Ten thousand HMCLs are plated into a 35 mm tissue culture plate for layer agar cultures and cultured for 21 days. Picture of one representative experiment were presented.

Figure S2

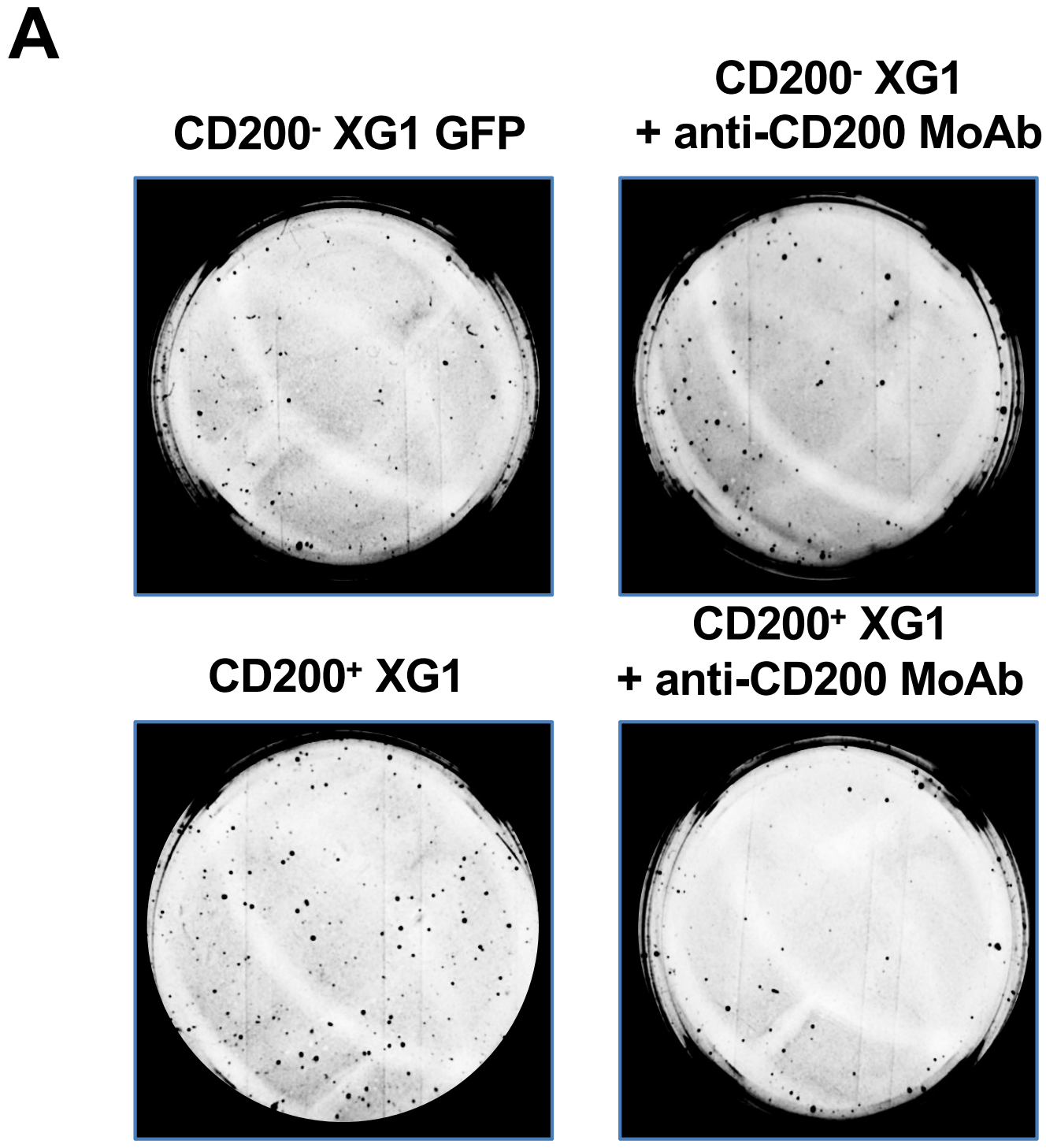

Supplementary Figure S2: (A) Clonogenic assays were performed with a collagen semi solid culture medium without cytokines (Stem Alpha III). Ten thousand HMCLs are plated into a 35 mm tissue culture plate for layer agar cultures and cultured for 21 days. Clonogenic assays were performed with or without a control MoAb (4µg/mL) or anti-CD200 MoAb (4µg/mL). Antibodies are added twice a week. Picture of one representative experiment were presented.

Figure S3

A

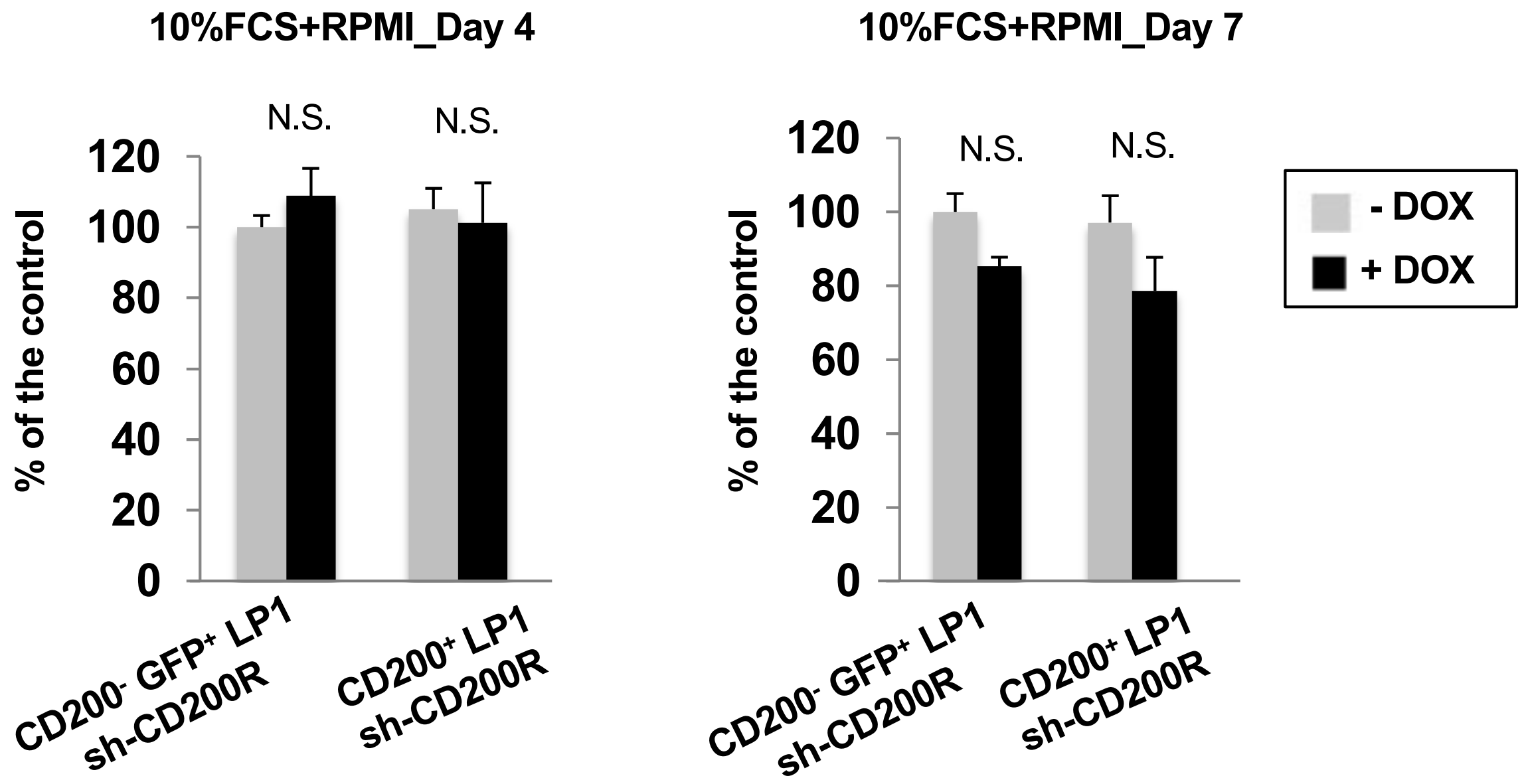

Supplementary Figure S3: (A) The effects of inducible depletion of CD200R (+ Doxycycline) in LP1CD200 negative cell line or in LP1CD200 positive cell line was investigated with cell titer glo assay in RPMI640 supplemented with 10% FCS. Results are the mean percentages (+/- SD) of the luminescent signal of each group compared to the control (LP1_shCD200R without doxycycline) in 3 independent experiments including sixplicate culture wells. *P* values were calculated using a Wilcoxon test for pairs. NS: not significant.

Figure S4

A

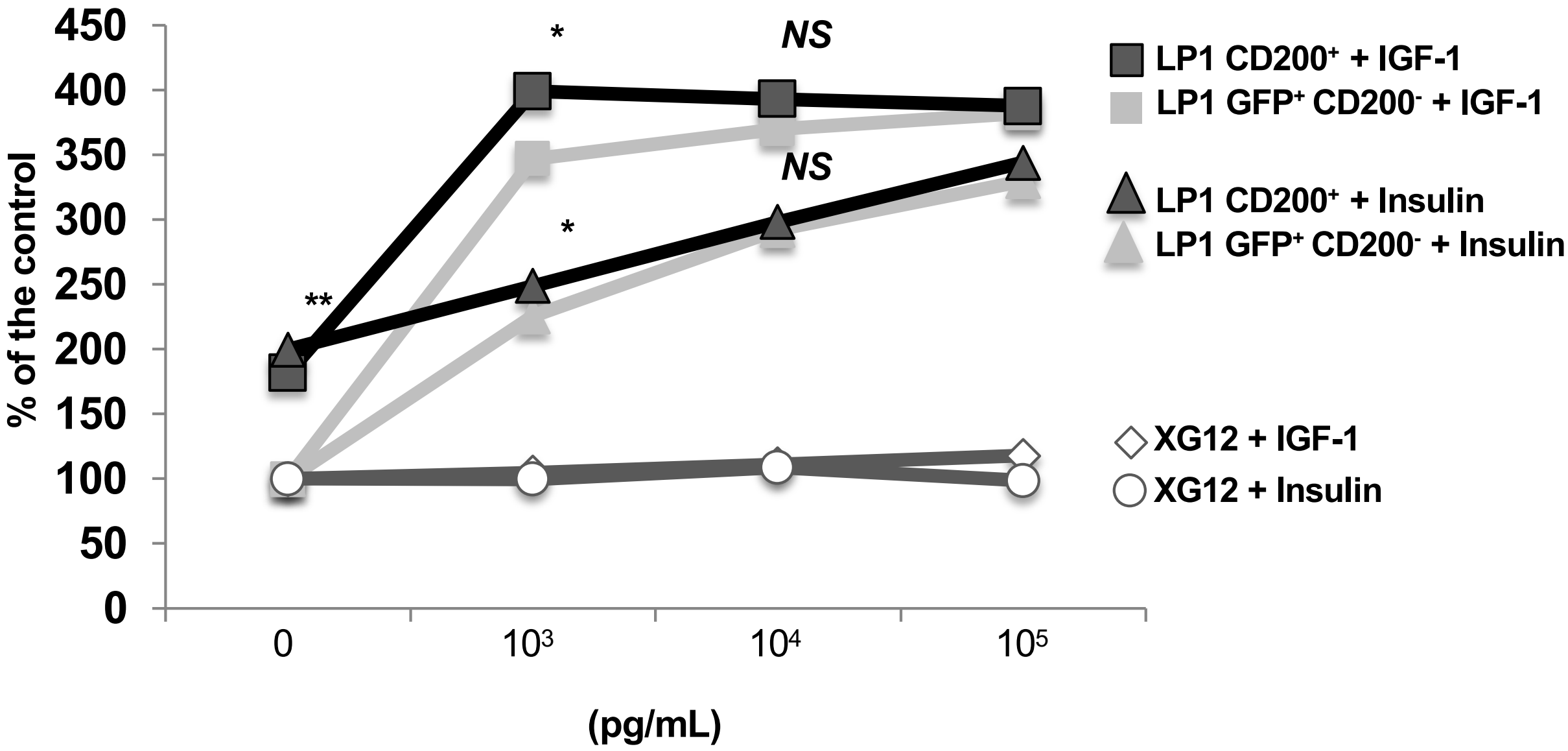

Supplementary Figure S4: (A) HMCLs were starved for 2 hours in serum free culture medium and cultured for 4 days in SynH serum free culture medium with or without different concentrations of IGF-1 or insulin. Cell growth was monitored using cell titer glo assay. *P* values were calculated using a Wilcoxon test for pairs (*P <0.05). NS: not significant.

Figure S5

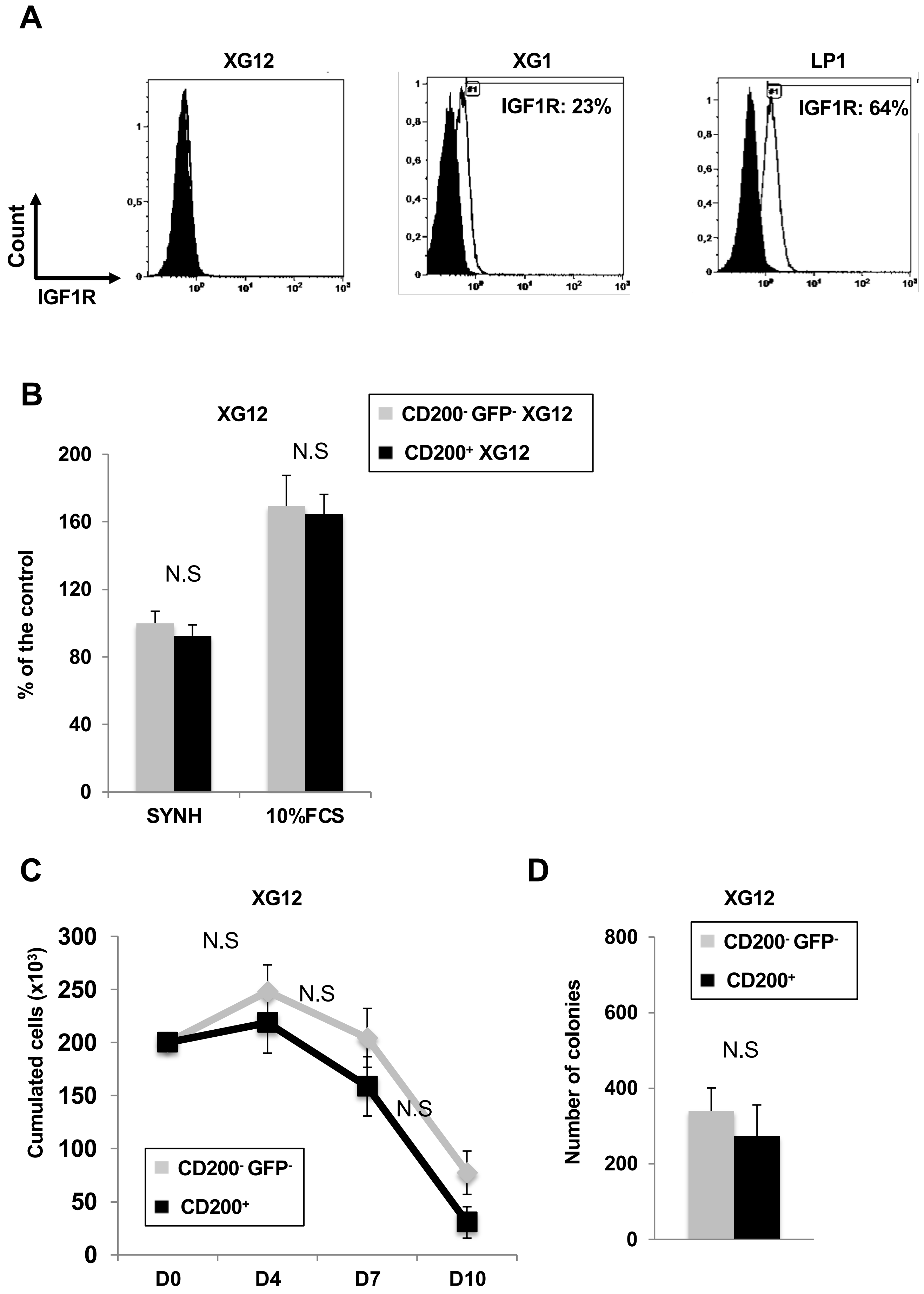

A
XG12
XG1
LP1
IGF1R: 23%
IGF1R: 64%
Count
IGF1R
B
XG12
CD200- GFP- XG12
CD200+ XG12
% of the control
N.S
N.S
SYNH
10%FCS
C
XG12
Cumulated cells (x10³)
N.S
N.S
N.S
CD200- GFP-
CD200+
D0
D4
D7
D10
D
XG12
Number of colonies
CD200- GFP-
CD200+
N.S

Supplementary Figure S5: (A) IGF1R protein expression was investigated in XG12, XG1 and LP1 HMCLs by flow cytometry. (B) XG12 GFP and XG12 CD200 were starved for 2 hours in serum free culture medium and cultured for 4 days in SynH serum free culture medium or RPMI1640 supplemented with 10% FCS. Cell growth was monitored using cell titer glo assay. *P* values were calculated using a Wilcoxon test for pairs. NS: not significant. (C) XG12 GFP and XG12 CD200 were starved for 2 hours in serum free culture medium and cultured for 10 days in SynH serum free culture medium. Cumulated cell growth was quantified. *P* values were calculated using a Wilcoxon test for pairs. NS: not significant. (D) Clonogenic assays were performed with a collagen semi solid culture medium without cytokines (Stem Alpha III). Ten thousand HMCLs are plated into a 35 mm tissue culture plate for layer agar cultures and cultured for 21 days. Results are the number of counted colonies (+/- SD) in each plate of 3 independent experiments. *P* values were calculated using a Wilcoxon test for pairs. NS: not significant.

Figure S6

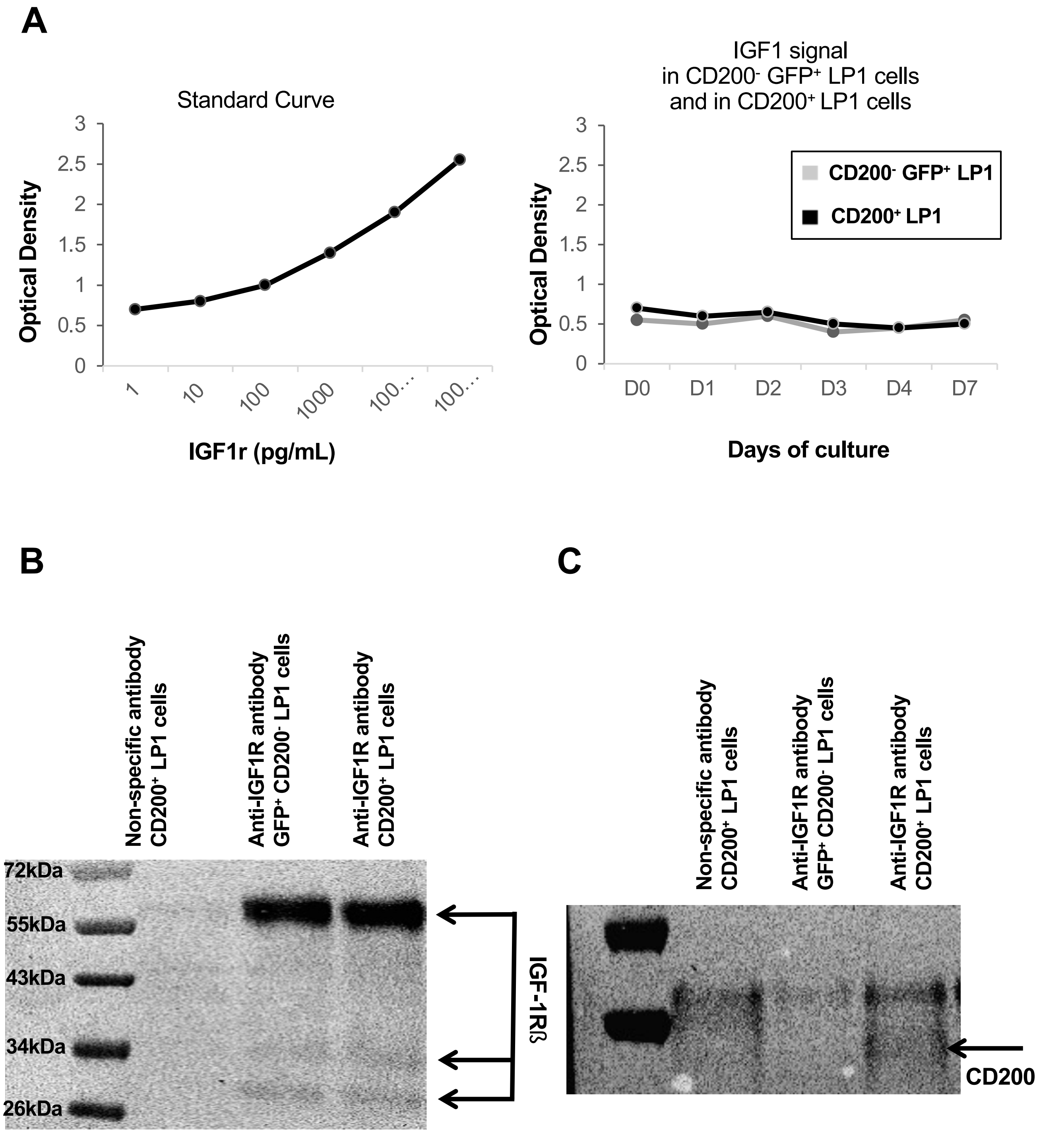

Supplementary Figure S6: (A) LP1GFP and LP1CD200 are cultured for 7 days in Syn H serum-free culture medium without cytokine and production of IGF-1 in the culture supernatant is measured using enzyme-linked ummunosorbent assay (ELISA) kit with a detection level of 50pg/mL. (B) The detection of IGF-1R in IP lysates (LP1 cell line) was assessed by western blot with anti IGF-IRß antibody (C-20, sc713, Santacruz). (C) CD200 was detected by western blot using anti-CD200 MoAb (AF2724, Bio-Techne).

Figure S7

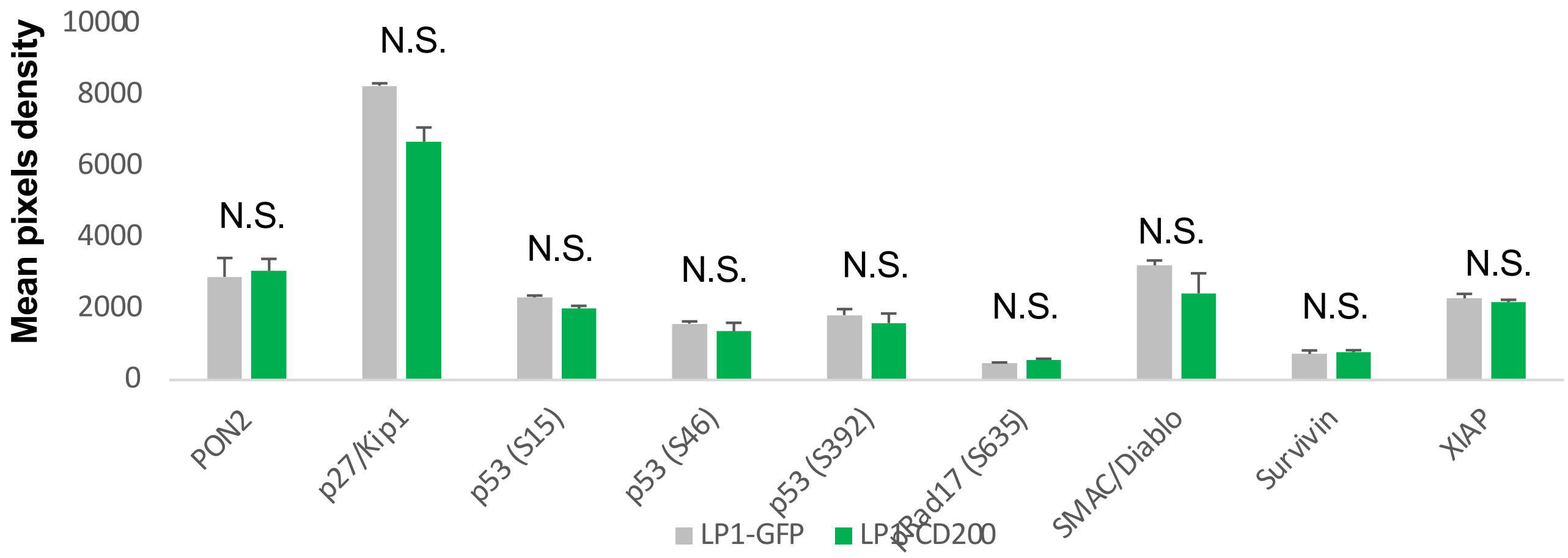

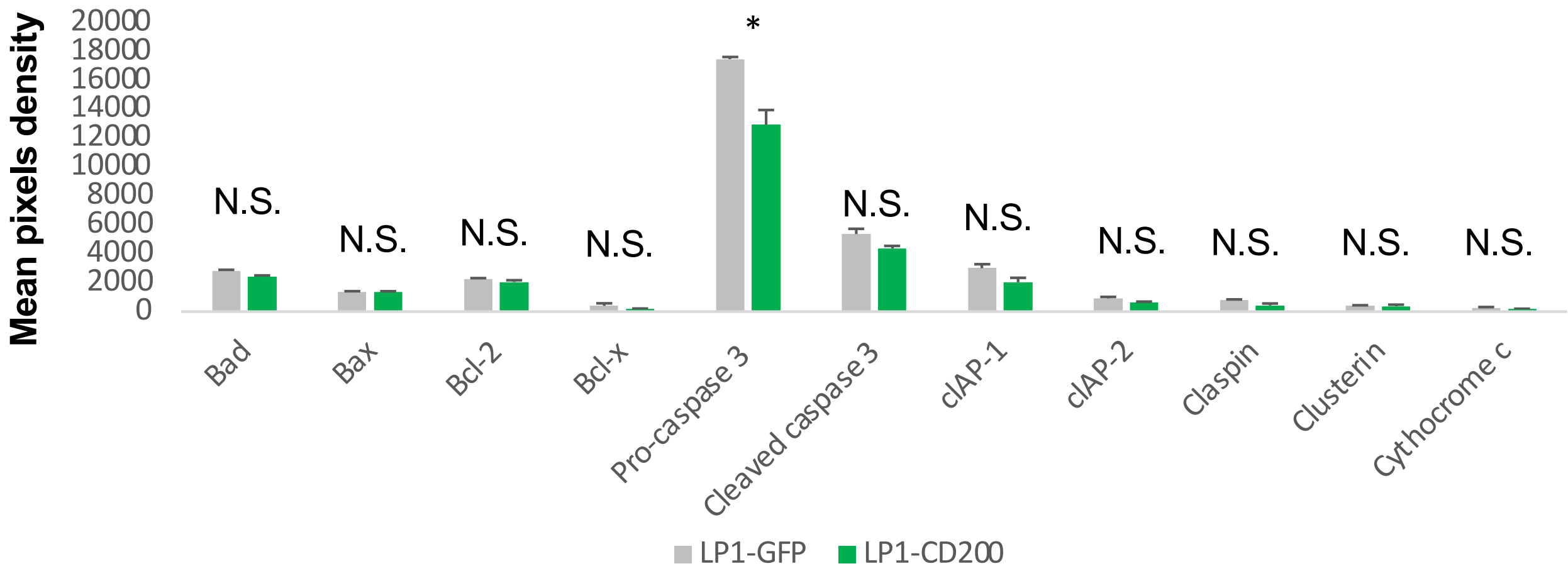

Supplementary Figure S7: Apoptosis and signaling pathways activated by CD200 expression in LP1 MM cell line. Proteins accumulations were monitored after 4 days of culture in SynH serum free culture medium using LP1 GFP control or CD200^{positive} HMCLs and proteome profiler arrays as previsously described[44]. Relative amount was calculated as the mean of pixel density. *P*-value: * *P* <0.05. NS: Not significant.
